# Effects of Nicotinamide Mononucleotide on Glucose and Lipid Metabolism in Adults: A Systematic Review and Meta-analysis of Randomised Controlled Trials

**DOI:** 10.1007/s11892-024-01557-z

**Published:** 2024-11-12

**Authors:** Feng Chen, Disheng Zhou, Alice Pik-Shan Kong, Nga Ting Yim, Siyu Dai, Yu Nan Chen, Lai Ling Hui

**Affiliations:** 1 School of Nursing, Li Ka Shing Faculty of Medicine, The University of Hong Kong, Hong Kong SAR, China; 2 Department of Medicine and Therapeutics, Faculty of Medicine, The Chinese University of Hong Kong, Hong Kong SAR, China; 3 Department of Applied Biology and Chemical Technology, The Hong Kong Polytechnic University, Hong Kong SAR, China; 4 Department of Paediatrics, Faculty of Medicine, The Chinese University of Hong Kong, Hong Kong SAR, China; 5 School of Clinical Medicine, Hangzhou Normal University, Hangzhou, China; 6 The Affiliated Hospital of Hangzhou Normal University, Hangzhou, China; 7 Department of Food Science and Nutrition, The Hong Kong Polytechnic University, Hong Kong SAR, China; 8 Research Institute for Future Food, The Hong Kong Polytechnic University, Hong Kong, China

**Keywords:** Nicotinamide mononucleotide, NAD, Supplementation, Glucose control, Lipid profile

## Abstract

**Purpose of Review:**

Supplementation of nicotinamide mononucleotides (NMN) has been claimed to improve metabolic function. We reviewed human randomised controlled trials (RCTs) of NMN to evaluate its effect on markers of glucose and lipid metabolism.

**Recent Findings:**

Eight RCTs on NMN (dosage ranged 250–2000 mg/d for a duration of 14 days to 12 weeks) involving a total of 342 middle-age/older adults (49% females, mainly non-diabetic) reporting at least one outcome on glucose control or lipid profile published in 2021–2023 were reviewed. The random-effects meta-analyses indicated no significant benefit of NMN on fasting glucose, fasting insulin, glycated hemoglobin, homeostatic model assessment for insulin resistance and lipid profile.

**Summary:**

Based on the small number of RCTs involving mainly relatively healthy adults, short-term supplementation of NMN of 250-2000 mg/d did not show significantly positive impacts on glucose control and lipid profile.

**Supplementary Information:**

The online version contains supplementary material available at 10.1007/s11892-024-01557-z.

## Introduction

Nicotinamide adenine dinucleotide (NAD +) is an important coenzyme of redox reactions involving in energy-generating pathways like β-oxidation, tricarboxylic acid cycle, and glycolysis. [1, 2] NAD + level decreases with age in mammals, [3, 4] and its depletion may be linked to age-related diseases, such as Alzheimer’s disease, type 2 diabetes, hyperglycaemia and cardiovascular disease. [5, 6] Nicotinamide mononucleotide (NMN), a precursor of NAD + , is an emerging supplement that has been extensively promoted for the claimed anti-ageing benefits including weight loss, enhanced metabolism [6] and prevention of age-related diseases, including type 2 diabetes and hyperglycaemia [7, 8].

Early in vivo studies demonstrated intraperitoneal administration of NMN improved glucose intolerance and lipid profiles in old and/or diabetic mice potentially through the NAD + /SIRT1 pathway. [9, 10] Orally administrated NMN (300 mg/kg body weight/day) for 12 months has been shown to enhance energy metabolism, higher physical activity, insulin sensitivity and lipid profiles in 5-month old mice. [11] Studies in human showed oral intake of NMN increases NAD + concentrations in the blood. However the uptake pathway of NMN in cells and tissues is still controversial, [12, 13] and thus it is not clear whether oral supplementation of NMN in humans improves glucose control and lipid profile. A recent comprehensive review and meta-analysis of 40 clinical studies related to the supplementation of different NAD + precursors and their effects on glucose and lipid metabolism reported that administration of nicotinic acid (NA) significantly improved lipid profile and hyperglycemia in humans, whereas nicotinamide riboside (NR) had insignificant effects. [14] This review did not included clinical trials of NMN supplementation. Two NMN trials among diabetic women [15] and amateur runners [16] were reviewed in a meta-analysis on NAD + precursors focusing on improvement of physical performance and frailty and both trials reported a null effect on markers of physical performance [17].

Here we conducted a systematic review and meta-analysis to evaluate the effects of NMN on markers of glucose and lipid metabolism compared with placebo or no intervention in randomised controlled trials (RCTs) in humans.

## Methods

### Overview

This systematic review and meta-analysis on RCTs of NMN supplementation was conducted and reported according to the guidelines from the updated Preferred Reporting Items for Systematic Reviews and Meta-Analyses (PRISMA) 2020 statement. [18] We sought to assess the effect of NMN on markers of glucose and lipid metabolism from RCTs compared with placebo or no intervention in humans. The protocol of this systematic review was registered at PROSPERO (CRD42022380334).

### Data Sources and Searches

We searched for relevant NMN trials from databases of PubMed, Web of Science, Embase, Scopus and Google Scholar using search terms (nicotinamide mononucleotide) AND (randomised controlled trial) from database inception through 31 May 2023. We also performed an additional search in ClinicalTrials.gov for unpublished relevant NMN trials that were registered and completed.

### Study Selection

We included trials on NMN supplement fulfilling the following criteria:

- **Type of studies**: RCT on human.
- **Population**: Adults aged 18 years or above.
- **Types of intervention**: NMN supplement of any duration.
- **Comparator**: Placebo or no interventions.
- **Outcome measures**: At least one biomarker related to glucose metabolism or lipid profile was assessed.

We excluded animal studies, in vitro studies, reviews, letters, comments, case reports and study protocols. We also excluded publications that were not written in English and publications that were not accessible in full-text format. The search and selection of literature, quality assessment and data extraction were performed independently by two co-authors (C.F. and Z.D.). Disagreements were discussed and resolved among C.F., Z.D. and two corresponding authors.

### Outcomes of Interest

We primarily evaluated outcomes related to glucose metabolism (fasting glucose, fasting insulin, glycated hemoglobin (HbA1c) and homeostatic model assessment for insulin resistance (HOMA-IR)) or lipid profile (total cholesterol, low-density lipoprotein (LDL) cholesterol, high-density lipoprotein (HDL) cholesterol and triglycerides. We also evaluated outcome related to liver function, body mass index (BMI) and blood pressure if the included study reported them.

### Data Extraction

The following information was extracted from each included study: (1) basic study information including first author, year of publication, study location, sample size and participants’ characteristics; (2) interventions: mode of administration, dosage of the supplementation (mg/d) and intervention duration; (3) measurements of outcomes at baseline and at endpoint; or the before and after trial difference; (4) safety related information, including serious adverse events, adverse events, markers of liver function (including aspartate transaminase (AST), alkaline phosphatase (ALP) and alanine transaminase (ALT)), BMI and blood pressure, and (5) funding for trials. We contacted authors for data that presented in plots, but we did not receive any response.

### Quality Assessment

The risk of bias was assessed by 2 reviewers (C.F. and Z.D.) using the Cochrane Risk of Bias Tool version 2 (Rob2) [19], which included the bias from randomization process, deviations from intended interventions, missing outcome data, measurement of the outcome and selection of the reported results. The discrepancies were discussed and resolved among C.F. and Z.D.

### Statistical Analyses

To conduct meta-analyses on markers of glucose and lipid profiles, the standardized mean difference (SMD) of each measure was calculated. SMD was calculated as the net difference of the change (endpoint – baseline) between intervention and control groups divided by the standard deviation (SD) of the change. We calculated the SD by using the following equation [20] if it was not provided in the study:

$$SD=\sqrt{{SD}_{baseline}^{2}}+{SD}_{endpoint}^{2}-2\times R\times {SD}_{baseline}\times {SD}_{endpoint}$$

where $$R=0.5$$

Random effects model (DerSimonian and Laird method [21]) was adopted to minimize the influence of heterogeneity of studies. I^2^ > 50% indicates substantial heterogeneity. [22] Tau2 was used to quantify the variance of the effect sizes. Forest plots were used to present the effect size of included studies. Review Manager 5.4 was used for meta-analysis and test of heterogeneity; alpha level was set at 0.05.

## Results

### Search Results

**Fig. 1:**
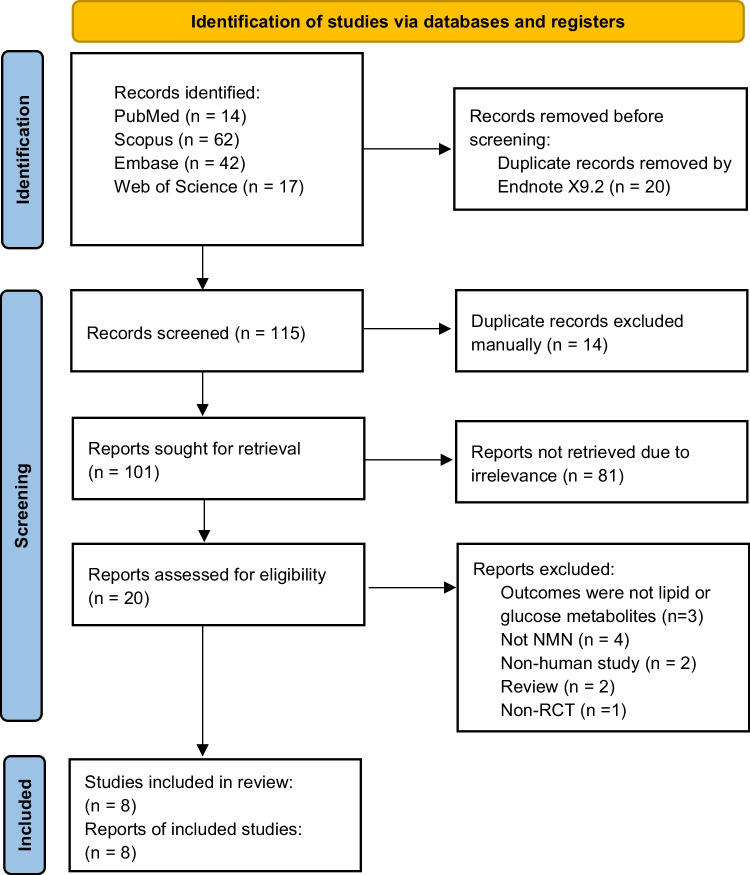
PRISMA diagram

After removal of duplicates, the initial search yield 101 unique articles from PubMed, Scopus, Embase and Web of Science (Fig. 1). Of these, 81 were found to be irrelevant reports after screening the title and abstract. Among the remaining 20 full-text articles, a total of 8 met eligibility criteria and included in this review.

Further search in ClinicalTrials.gov found another 16 NMN trials (6 completed; 6 recruiting, 1 active but not recruiting, 2 not yet recruiting, 1 unknown status) examining the effect of NMN on at least one marker of glucose control or lipid profile as outcome measures (Supplementary Table 1). However, none of the completed trials reported their results in the registry and thus we were not able to include any in this review.

### Characteristics of Included RCTs

All 8 eligible studies were double-blinded, randomised and controlled trials, among which 2 trials [23, 24] had randomised design with mutiple intervention groups (Table 1). All trials were reported during 2021–2023, and the majority were conducted in Asia. The funding source for these trials was summarised in Supplementry Table 2.

**Table 1:** Study design and participant baseline characteristics of the included RCTs

Study	Country	Participant	Duration	Group	Age (yrs)	Gender	Baseline Measurements								
							BMI	Fasting glucose (mg/dL)	HbA1c (%)	HOMA-IR	Insulin (μU/mL)	Triglycerides (mg/dL)	LDL cholesterol (mg/dL)	HDL cholesterol (mg/dL)	Total cholesterol (mg/dL)
Katayoshi et al., 2023 [27]	Japan	36 amateur runners	12 weeks	NMN (250 mg/d)	48.1 ± 5.4	8 M + 10F	21.9 ± 4.3	87.0 ± 4.5	-	-	-	69.1 ± 35.9	119.9 ± 25.9	72.2 ± 15.5	-
				Placebo	47.9 ± 5.5	6 M + 12F	21.7 ± 2.3	90.1 ± 5.9	-	-	-	86.4 ± 54.7	122.7 ± 54.1	73.5 ± 16.5	-
Yi et al., 2023[24]	India	80 adults	60 days	NMN (900 mg/d)	49.9 ± 6.3	9 M + 11F	26.9 ± 4.9	-	-	2.0 ± 1.3	-	178 ± 83	132.8 ± 39.7	40.5 ± 8.2	190 ± 47
				NMN (600 mg/d)	49.5 ± 6.7	6 M + 14F	27.1 ± 3.9	-	-	1.7 ± 1.1	-	157 ± 62	119 ± 22	41.8 ± 5.6	179 ± 26
				NMN (300 mg/d)	51.2 ± 7.0	10 M + 10F	27.4 ± 4.8	-	-	2.3 ± 1.4	-	159 ± 70	122 ± 32	43.8 ± 12.3	176 ± 32
				Placebo	46.5 ± 6.7	8 M + 12F	26.9 ± 4.9	-	-	1.4 ± 0.8	-	156 ± 86	114 ± 30	42.4 ± 10.0	171 ± 32
Huang, 2022[26]	India	66 adults	60 days	NMN (300 mg/d)	47.8 ± 6.6	13 M + 18F	25.3 ± 2.3	95.3 ± 21.0	-	1.8 ± 0.9	14.3 ± 6.8	141.0 ± 77.5	115.5 ± 26.1	-	178.6 ± 38.9
				Placebo	47.2 ± 6.6	15 M + 16F	24.7 ± 2.4	101.9 ± 27.0	-	1.8 ± 1.2	15.1 ± 9.3	168.5 ± 106.3	124.7 ± 42.1	-	186.2 ± 50.7
Fukamizu et al., 2022[29]	Japan	31 adults	4 weeks	NMN (1250 mg/d)	35.1 ± 7.0	7 M + 9F	22.9 ± 2.7	91.3 ± 4.1	-	-	7.1 ± 3.8	97.9 ± 57.8	122.3 ± 34.8	66.3 ± 13.6	209.1 ± 38.6
				Placebo	35.7 ± 7.2	7 M + 8F	22.1 ± 3.3	93.5 ± 8.8	-	-	6.9 ± 2.9	77.5 ± 47.0	121.9 ± 24.8	68.6 ± 19.2	209.3 ± 28.9
Igarashi et al., 2022[25]	Japan	42 adults	12 weeks	NMN (250 mg/d)	71.1 ± 3.9	21 M	24.1 ± 1.4	99.0 ± 9.0	5.85 ± 0.58	1.4 ± 0.8	-	116.7 ± 36.8	125.4 ± 32.3	67.3 ± 17.9	-
				Placebo	71.8 ± 6.1	21 M	24.5 ± 1.4	94.4 ± 7.8	5.74 ± 0.32	1.1 ± 0.4	-	102.2 ± 44.9	132.2 ± 18.0	61.1 ± 15.2	-
Okabe et al., 2022[28]	Japan	30 adults	12 weeks	NMN (250 mg/d)	42.9 ± 12.0	4 M + 11F	21.3 ± 2.5	97.5 ± 5.1	5.37 ± 0.23	-	-	66.7 ± 19.2	105.2 ± 24.7	-	-
				Placebo	43.9 ± 9.9	4 M + 11F	21.1 ± 2.1	98.4 ± 6.5	5.38 ± 0.31	-	-	66.5 ± 19.4	106.6 ± 17.3	-	-
Pencina et al., 2023[23]	USA	32 adults	14 days	NMN (2000 mg/d)	64.0 ± 5.6	6 M + 6F	27.7 ± 2.5	94.2 ± 10.4	-	-	-	107.0 ± 56.7	110.5 ± 23.4	53.1 ± 14.8	84.2 ± 31.4
				NMN (1000 mg/d)	62.4 ± 6.1	6 M + 6F	30.7 ± 3.2	93.6 ± 10.2	-	-	-	99.9 ± 41.8	110.5 ± 21.7	49.3 ± 12.5	179.8 ± 31.2
				Placebo	66.2 ± 7.0	4 M + 4F	28.7 ± 1.9	94.1 ± 5.9	-	-	-	123.9 ± 83.6	111.1 ± 27.4	52.3 ± 15.1	184.8 ± 34.6
Yoshino et al., 2021[15]	USA	25 prediabetic women	10 weeks	NMN (250 mg/d)	62.0 ± 4.0	12F	33.7 ± 1.4	87.0 ± 4.5	5.7 ± 0.1	-	13.6 ± 1.9	1.39 ± 0.25	-	1.25 ± 0.10	-
				Placebo	61.0 ± 5.0	13F	33.4 ± 1.0	90.1 ± 5.9	5.5 ± 0.1	-	16.7 ± 2.0	1.63 ± 0.22	-	1.28 ± 0.07	-

Abbreviations: NMN: nicotinamide mononucleotide; M: male; F: female; AM: morning; PM: afternoon; HbA1c: haemoglobin A1c; HOMA-IR: homeostatic model assessment of insulin resistance; LDL: low-density lipoprotein cholesterol; HDL: high-density lipoprotein cholesterol

All included trials were small in scale, with sample size ranging from 14 to 108, and a total of 342 participants (49% female) took part. The trials were mainly carried out among middle-aged adults except 3 RCTs recruited older subjects with mean age over 60 years [15, 23, 25]. Most trials recruited subjects with normal baseline glucose and lipid profiles except 1 study recruited prediabetic patients. [15] 4 RCTs were conducted among subjects with mean BMI of 25 or above. [15, 23, 24, 26]

NMN supplements were orally administrated in all trials. Duration of supplementation ranged from 14 days to 12 weeks, with 6 trials [15, 24–28] having intervention more than 8 weeks. The dosage of NMN ranged from 250 mg/d to 2000 mg/d; 250 mg/d was the most commonly used dosage and was employed in 4 studies. [15, 25, 27, 28] The largest dosage of 2000 mg/d was tested in Pencina et al.,2023 for 14 days. [23]

The majority of included trials tested fasting glucose [15, 23, 25–29] and/or lipid profile. [23–29] Some studies also evaluated the effect of NMN on fasting insulin [15, 26, 28, 29], HOMA-IR [24–26, 28] and HbA1c [15, 25, 28].

### Quality Assessment

**Fig. 2:**
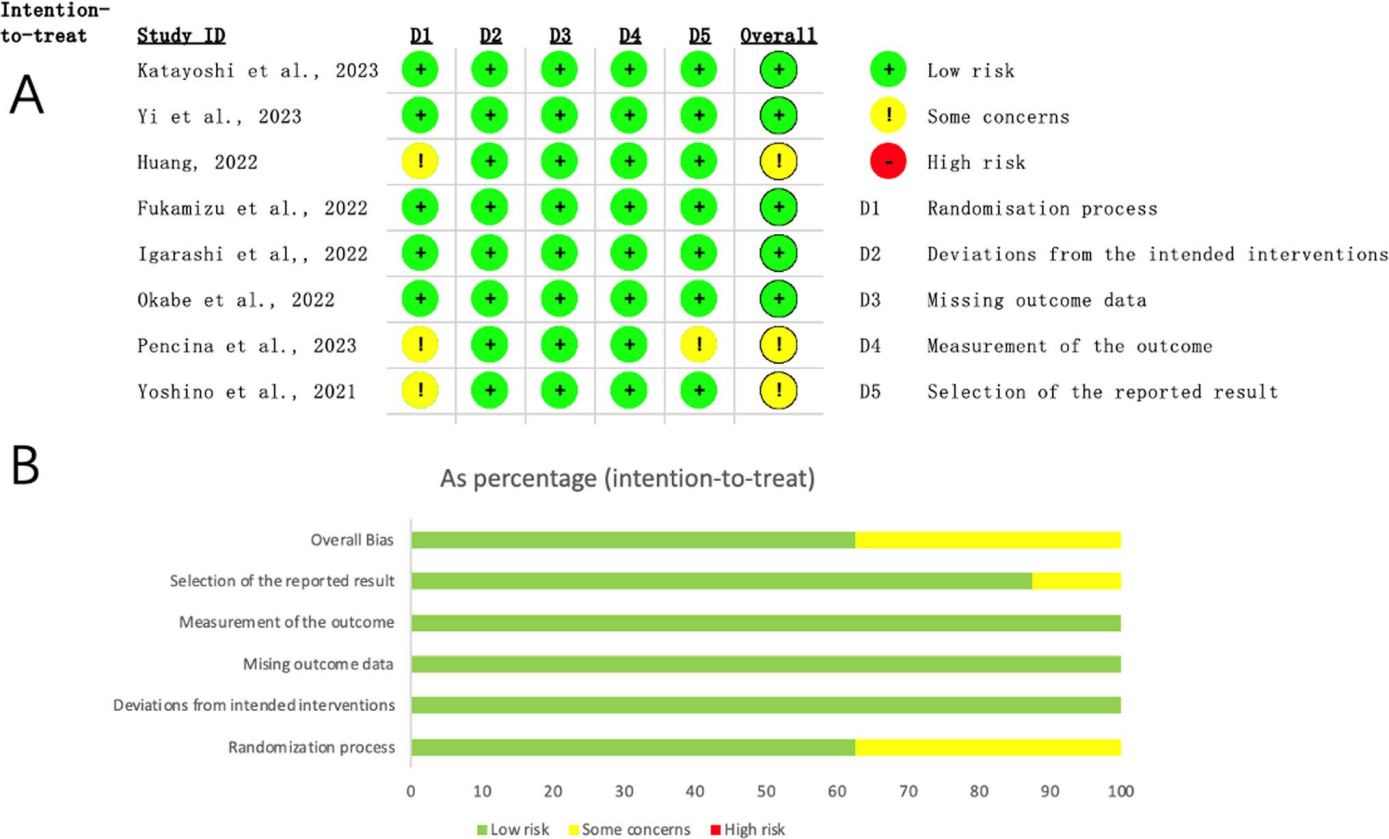
Risk of bias assessment in the randomised parallel-arm studies. **A**. Risk of bias summary. **B**. Quality assessment percentages of all included studies

Based on an adapted version of Rob2 implemented specifically for parallel-group trials with individual randomization (Fig. 2), 5 studies had a low risk of bias. [24, 25, 27–29] The other 3 studies had some concerns about the overall risk of bias due to lacking information of allocation concealment in the randomization process [15, 23, 26] or analysis plan for the selection of the reported result [23].

### Effects of NMN on Markers of Glucose and Lipid Metabolism

**Fig. 3:**
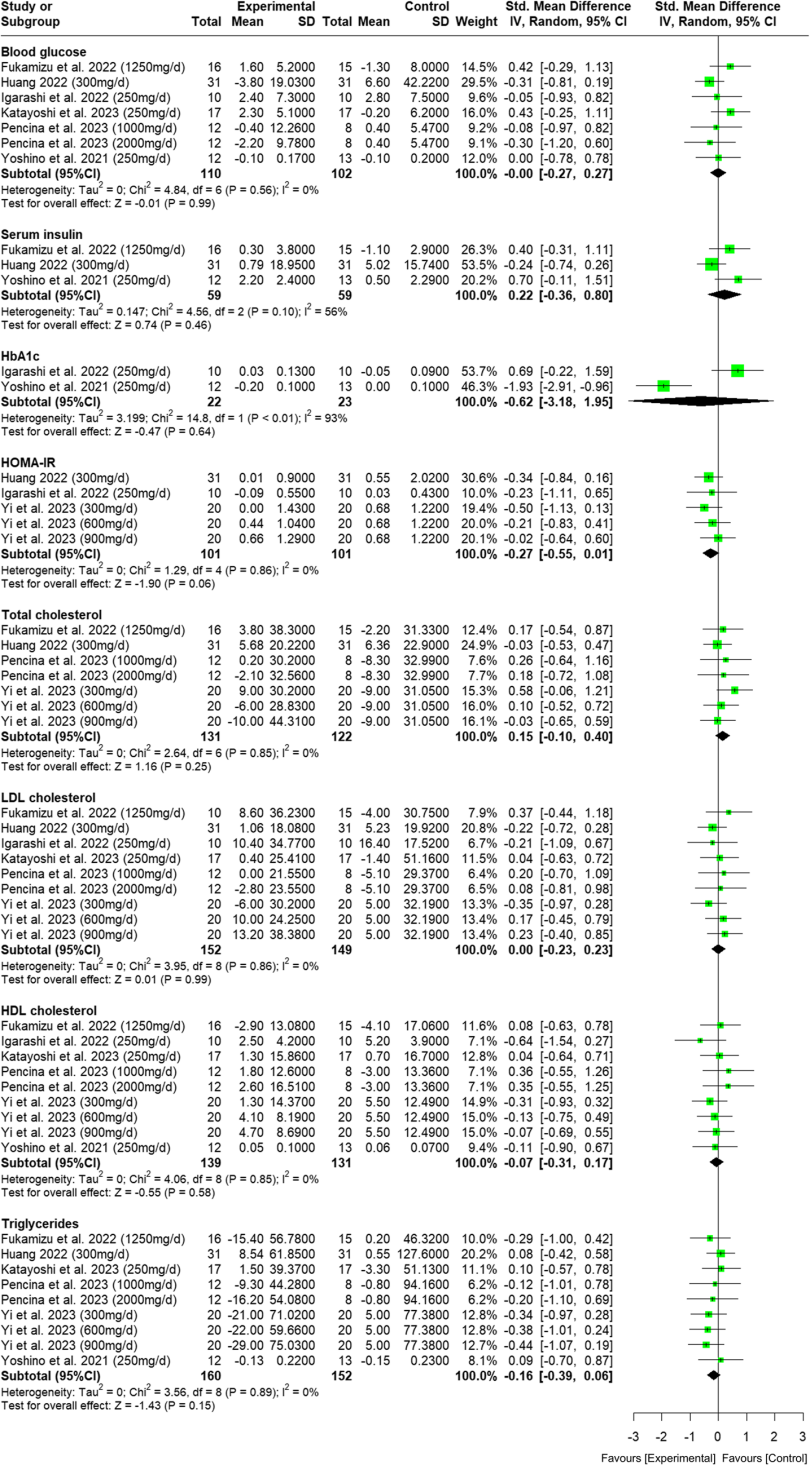
Forest plot of the effect of NMN supplements on markers of glucose and lipid metabolism

Five out of the 8 RCTs reported an increase in blood NAD + level following NMN supplementation. [23–27] The meta-analyses indicated no significant benefit of NMN on fasting glucose, fasting insulin, HbA1c and lipid profile. There was a marginally significant reduction on HOMA-IR by 0.27 (95% CI -0.01 to 0.55; p = 0.06; I^2^ = 0%) based on 3 studies (Fig. 3). However this effect on HOMA-IR became not signficant after excluding the trial by Huang 2022 [26] whose mean difference in HOMA-IR was attributed to a deterioration of HOMA-IR in placebo group, instead of an improvement in the supplementation group. Included studies had low heterogeneity (I^2^ = 0%) for almost all outcome measures except for fasting insulin and HbA1c with I^2^ > 50%.

Okabe et la., 2022, [28] presented their results in plots only thus its data was not included in the meta-analyses. It reported null difference in the related biomarkers between NMN group and placebo group from a small sample of 30 subjects, therefore including its data in the meta-analysis would unlikely affect the combined results. Given none of meta-analyses included findings from 10 or more trials, we are unable to examine from a funnel plot to explore publication bias.

### Safety of NMN

Reports of adverse events in the included trials were summarised. (Supplementary Table 3) Three trials reported no adverse events. No trial reported safety concerns of NMN supplementation, however there seems to be a tendency of more adverse events, regardless of their relevance to the intervention, reported in NMN groups with dosage 1000 mg/d or higher. [23, 29] Additional meta-analyses found no statistically significant difference in BMI, blood pressure and liver function (AST, ALT and ALP levels) in 6 studies [15, 24–27, 29] between the NMN intervention groups (250–1250 mg/d) and the controls. (Supplementary Fig. 1).

## Discussion

This systematic review of 8 small-scale RCTs found short-term NMN supplementation did not improve markers of glucose control and lipid profile. The majority of them included subjects with normal baseline glucose and lipid profiles, as such our findings mainly apply to the general population without impaired glucose control or lipid profile.

Restoring the NAD + level by NMN has been suggested to ameliorate age-associated metabolic dysfunction [30] with beneficial effects on glucose and lipid metabolism shown in rodent disease models. The lack of benefits of NMN supplementation in humans reviewed here may be due to some key differences between mice models and human studies. Firstly, NMN was injected intraperitoneally in some early mice studies which showed NMN improved glucose intolerance and lipid profiles, [9, 10] while NMN human trials use oral NMN supplementation. It has been demonstrated using isotope-tracer methods that intravenous administration of NMN delivered intact molecules to multiple tissues while at least some of the orally delivered NMN are converted to nicotinamide before reaching the systemic circulation. [31] Although 5 included NMN trials reported an increase in blood NAD + following oral supplementation of NMN, [23–27] it is not clear whether this translated to cellular level activity of NAD + , which is likely to be tissue-specific. [31] Secondly, the dosage of NMN used in the included human RCTs ranged from 250 mg/d to 2000 mg/d, which is equivalent to about 4–33 mg/kg body weight/d assuming a body weight of 60 kg. The NMN dosage that exerted a beneficial effect on glucose and lipid metabolism in mice models was as high as 300 mg/kg body weight/d orally for months [11] or 500 mg/kg body weight/d by injection for 18 days [32].

Despite it is possible the NMN doses in human studies were too low compared to animal studies that demonstrated its beneficial effects on metabolic outcomes, the unintended potential side-effects of high NMN dosage in humans require careful considerations. Given orally administrated NMN can be converted to nicotinamide, the upper limit of nicotinamide (900 mg/d) and its link with cardiovascular disease risk [33] need to be carefully considered for higher supplementation dose of NMN. Another concern is the worsening senescence-associated secretory phenotype and tumorigenesis by an increase in NAD + level. [34] The effect of NMN supplement is likely complex and to be age- as well as tissue- specific, with beneficial and adverse effects of NMN supplementation observed in different organs. [35] None of the included NMN human trials in this systematic review reported severe adverse effects, but the NMN supplementation was mainly 12 weeks or shorter. The safety of long-term supplementation of a higher dosage of NMN in humans, particularly among the old age, is uncertain and should be considered cautiously.

The lack of benefits from NMN supplementation in our meta-analyses were mainly consistent with another recent meta-analysis on other NAD + precursors [14] which showed similar null findings of NR supplementation on markers of glucose and lipid metabolism summarized from 3 studies. [36–38] NR is another vitamin B3 derivative that is involved in NAD + biosynthesis and it also closely links with NMN. Human trials on NR supplementation more recently published similarly reported no benefits of NR on glucose control [39–42] and lipid profile [39] in fasting blood, hepatic insulin sensitivity [39] as well as glucose uptake [40] and lipid deposition [43] in skeletal muscle. In mammals, NMN is synthesized from NR via an NR kinase-mediated reaction. NMN can also be metabolized extracellularly by NR kinase 1 to NR which is then taken up by the cell and converted to NAD + . [12] Similar to NMN, orally administrated NR is mainly metabolized to nicotinamide in the liver instead of entering the circulation intact. It has even been suggested that the effect of orally ingested NR and NMN would be similar to effect of oral administration of nicotinamide. [31] The uptake pathway of orally administrated NMN and NR from the gut to tissue casts doubt on the effectiveness of their supplementations in restoring the NAD + activities at the cell level.

This systematic review has some limitations. Although most of the included trials were classified as low risk of bias based on the Rob2, the number of trials was small and their sample sizes were also small. We were unable to reliably examine publication bias due to the paucity of data. Not all trials measured and reported all markers of glucose control and lipid profile and thus some combined results were only based on 2 to 3 studies. The dosage of NMN supplements, intervention periods and subject characteristics varied in the included trials, as such the combined effect of NMN is difficult to interpret. Only one included NMN trial recruited prediabetic participants which showed improved insulin sensitivity in muscle. While sub-group analysis by health status is not allowed, findings from current meta-analysis pooling study results from healthy subjects and diabetic subjects cannot be generalized to diabetic or hyperlipidemia patients or those who are at risk of these conditions. Further RCTs of both short- and longer-term NMN supplementation among patients with impaired glucose control or lipid profile with measurements of NMN uptake into blood and cells are warranted to clarify its NAD + boosting effect and pathway.

## Conclusions

This systematic review on 8 small-scale RCTs involving mainly relatively healthy adults did not find short-term NMN supplementation improved markers of glucose control and lipid profile. Our findings do not support the use of NMN supplementation among general population to improve glucose and lipid metabolism.

## Key References

- Yoshino M, et al. Nicotinamide mononucleotide increases muscle insulin sensitivity in prediabetic women. Science. 2021;372(6547):1224–1229.
  - The first randomised controlled trial and it showed NMN supplementation increases muscle insulin sensitivity and insulin signaling in women with prediabetes.
- Liu L, et al. Quantitative analysis of NAD synthesis-breakdown fluxes. Cell Metab. 2018;27(5):1067–1080 e5.
  - First comprehensive NAD flux analysis studying the NAD synthesis and breakdown pathway, revealing tissue-specific NAD metabolism. It showed orally administered NMN was metabolised to nicotinamide in the liver.
- Zhong O, et al. Effects of NAD+ precursor supplementation on glucose and lipid metabolism in humans: a meta-analysis. Nutr Metab (Lond). 2022;19(1):20.
  - The first systematic review on supplementing NAD+ precursors on glucose and lipid metabolism in humans but NMN was not included.

## Supplementary Information

Below is the link to the electronic supplementary material.

Supplementary file1 (DOCX 10.1 MB)

## Data Availability

No datasets were generated or analysed during the current study.

## Abbreviations

ALP: Alkaline phosphatase
ALT: Alanine transaminase
AST: Aspartate transaminase
BMI: Body mass index
CONSORT: Consolidated Standards of Reporting Trials
HbA1c: Glycated hemoglobin
HDL: High-density lipoprotein
HOMA-IR: Homeostatic model assessment for insulin resistance
LDL: Low-density lipoprotein
NA: Nicotinic acid
NAD +: Nicotinamide adenine dinucleotide
NMN: Nicotinamide mononucleotides
NR: Nicotinamide riboside
PRISMA: Preferred Reporting Items for Systematic Reviews and Meta-Analyses
SD: Standard deviation
SMD: Standardized mean difference
