# Supplementary material for: Effects of Nicotinamide Mononucleotide on Glucose and Lipid Metabolism in Adults: A Systematic Review and Meta-analysis of Randomised Controlled Trials
Source: Curr Diab Rep. 2024 Nov 12;25(1):4. doi: 10.1007/s11892-024-01557-z (PMC11557618; doi:10.1007/s11892-024-01557-z)

**Supplementary Table 1. Unpublished trials registered in ClinicalTrials.gov as of 20 Jun 2023 (Status updated on 28 Oct 2024)**

|  | **Title** | **Status** | **Study Results** | **Conditions** | **Interventions** | **Location** |
| --- | --- | --- | --- | --- | --- | --- |
| **1** | Pharmacodynamics and Tolerance of Nicotinamide Mononucleotide (NMN, 400mg/Day) in Healthy Adults | Completed | Not Available | • Healthy Volunteers | • Dietary Supplement: Nicotinamide mononucleotide (NMN-C) | France |
| **2** | Study to Evaluate the Effect of Nicotinamide Mononucleotide (NMN) As an Adjuvant to Standard of Care (SOC) On Fatigue Associated With COVID-19 Infection | Terminated | Not Available | • COVID-19 Infection | • Other: Nicotinamide Mononucleotide • Other: Nicotinamide Mononucleotide with L-Leucine • Other: Placebo | India |
| **3** | Nicotinamide Mononucleotide in Hypertensive Patients | Unknown status | Not Available | • Hypertension | • Dietary Supplement: Nicotinamide mononucleotide supplied as 400mg capsule • Behavioural: Lifestyle modification | China |
| **4** | Safety and Pharmacokinetics of Nicotinamide Mononucleotide (NMN) in Healthy Adults. | Completed | Not Available | • Healthy Volunteers | • Dietary Supplement: Nicotinamide mononucleotide (NMN-C) | Canada |
| **5** | Metabolic Changes Induced by NMN in Healthy Subjects With Acute Binge Drink | Active, not recruiting | Not Available | • Binge Drinking • Liver Injury • Nutritional Supplementation • Hepatic Steatosis | • Dietary Supplement: nicotinamide Mononucleotide • Dietary Supplement: Maltodextrin | China |
| **6** | Effect of NMN (Nicotinamide Mononucleotide) on Polycystic Ovary Syndrome | Recruiting | Not Available | • Polycystic Ovary Syndrome | • Dietary Supplement: NMN intervention • Other: Placebo | China |
| **7** | Effect of NMN (Nicotinamide Mononucleotide) on Diminished Ovarian Reserve (Including Premature Ovarian Insufficiency) | Recruiting | Not Available | • Diminished Ovarian Reserve • Premature Ovarian Insufficiency | • Dietary Supplement: NMN intervention • Other: Placebo | China |
| **8** | Effect of "Nicotinamide Mononucleotide" (NMN) on Cardiometabolic Function | Completed | Not Available | • Glucose Metabolism Disorders | • Dietary Supplement: NMN supplement • Other: Placebo | United States |
| **9** | Effect of NMN on Muscle Recovery and Physical Capacity in Healthy Volunteers With Moderate Physical Activity | Completed | Not Available | • Physical Activity • Muscle Recovery | • Dietary Supplement: Nicotinamide mononucleotide • Other: Placebo | France |
| **10** | NAD-brain: a Pharmacokinetic Study of NAD Replenishment Therapy | Recruiting | Not Available | • Healthy | • Dietary Supplement: nicotinamide riboside • Dietary Supplement: nicotinamide mononucleotide | Norway |
| **11** | Effects of MIB-626 With and Without A High-Intensity Multi- Dimensional Exercise Training Program | Recruiting | Not Available | • Healthy | • Drug: Investigational Product - MIB 626 • Drug: Placebo • Other: Standardized, progressive, high intensity, multidimensional exercise • Other: Usual Physical Activity | United States |
| **12** | To Evaluate the Efficacy and Safety of NMN as an Anti-ageing Supplement in Middle Aged and Older (40-65 Years) Adults | Completed | Not Available | • Aging | • Drug: Nicotinamide Mononucleotide • Other: Placebo | India |
| **13** | Evaluate the Efficacy and Safety of Uthever NMN(Nicotinamide Mononucleotide, a Form of Vitamin B3) | Completed | Not Available | • Aging | • Other: NMN • Other: Placebo | India |
| **14** | Effect of NMN Supplementation on Organ System Biology | Recruiting | Not Available | • Glucose Metabolism Disorders | • Other: Placebo • Dietary Supplement: Treatment | United States |
| **15** | Effect of Oral NAD+ Precursors Administration on Blood NAD + Concentration in Healthy Adults | Active, not recruiting | Not Available | • Healthy | • Dietary Supplement: Nicotinamide (NAM) • Dietary Supplement: Nicotinamide Riboside (NR) • Dietary Supplement: Nicotinamide Mono Nucleotide (NMN) • Dietary Supplement: Microcrystalline cellulose | Switzerland |
| **16** | NAD Augmentation in Diabetes Kidney Disease | Recruiting | Not Available | • Type 2 diabetes • Diabetic Kidney Disease | • Drug: Investigational Product - MIB 626 • Drug: Placebo | United States |

**Supplementary Table 2. Funding source of the included RCTs**

| **Study** | **Funding sources** |
| --- | --- |
| Katayoshi et al., 2023[27] | DHC Corporation |
| Yi et al., 2023[24] | Aba Chemicals Co., Ltd. (Shanghai, China) in collaboration with Abinopharm, Inc. (Connecticut, USA) |
| Huang, 2022[26] | No funding source was specified. The author’s affiliation is Effepharm (Shanghai) Co., Ltd. |
| Fukamizu et al., 2022[29] | Mitsubishi Corporation Life Sciences Limited |
| Igarashi et al., 2022[25] | Mitsubishi Corporation Life Sciences Limited |
| Okabe et al., 2022[28] | Mitsubishi Corporation Life Sciences Limited |
| Pencina et al., 2023[23] | Metro International Biotech |
| Yoshino et al., 2021[15] | National Institutes of Health SIG grant |

**Supplementary Table 3. Summary of adverse events.**

| **Study** | **N** | **Adverse events** | **Placebo** | **NMN dosage (mg/d)** | | | | | | |
| --- | --- | --- | --- | --- | --- | --- | --- | --- | --- | --- |
|  |  |  |  | **250** | **300** | **600** | **900** | **1000** | **1250** | **2000** |
| Katayoshi et al. 2023[27] | 36 | Any AEs | 0 | 0 | - | - | - | - | - | - |
| Yi et al., 2023[24] | 80 | Any AEs | 6 | - | 3 | 0 | 0 | - | - | - |
|  |  | Rashes on skin | 1 | - | 0 | 0 | 0 | - | - | - |
|  |  | Tingling and numbness in all extremities | 1 | - | 0 | 0 | 0 | - | - | - |
|  |  | Weakness of right upper extremity | 1 | - | 0 | 0 | 0 | - | - | - |
|  |  | Irrelevant talk | 1 | - | 0 | 0 | 0 | - | - | - |
|  |  | Mouth ulcer | 1 | - | 1 | 0 | 0 | - | - | - |
|  |  | Fever | 1 | - | 0 | 0 | 0 | - | - | - |
|  |  | Hyperacidity | 0 | - | 1 | 0 | 0 | - | - | - |
|  |  | Skin problem | 0 | - | 1 | 0 | 0 | - | - | - |
| Huang, 2022 [26] | 66 | Any AEs | 1 | - | 1 | - | - | - | - | - |
|  |  | Dyslipidemia | 1 | - | 1 | - | - | - | - | - |
| Fukamizu et al. 2022[29] | 31 | Any AEs | 1 | - | - | - | - | - | 4 | - |
|  |  | Loose stool | 1 | - | - | - | - | - | 1 | - |
|  |  | Common cold | 0 | - | - | - | - | - | 1 | - |
|  |  | High blood pressure | 0 | - | - | - | - | - | 1 | - |
|  |  | Acne vulgaris | 0 | - | - | - | - | - | 1 | - |
| Igarashi et al. 2022[25] | 42 | Any SAEs | 0 | 0 | - | - | - | - | - | - |
| Okabe et al. 2022[28] | 30 | Any AEs | (7)* | (8)* | - | - | - | - | - | - |
|  |  | Any SAEs | 0 | 0 | - | - | - | - | - | - |
|  |  | AEs due to intervention | 1 | 1 | - | - | - | - | - | - |
|  |  | ﻿Gastrointestinal symptoms | 2 | 3 | - | - | - | - | - | - |
|  |  | Fever, joint pain, or fatigue | 2 | 6 | - | - | - | - | - | - |
|  |  | ﻿Muscle pain | 2 | 2 | - | - | - | - | - | - |
|  |  | ﻿Upper respiratory tract symptoms | 1 | 1 | - | - | - | - | - | - |
|  |  | ﻿Hives | 0 | 1 | - | - | - | - | - | - |
|  |  | ﻿Headache | 2 | 0 | - | - | - | - | - | - |
|  |  | ﻿Keratitis, dry eye | 1 | 0 | - | - | - | - | - | - |
|  |  | Toothache | 1 | 0 | - | - | - | - | - | - |
| Pencina et al. 2023[23] | 32 | Any AEs | 18 | - | - | - | - | 40 | - | 31 |
|  |  | Any SAEs | 0 | - | - | - | - | 0 | - | 0 |
|  |  | Blood and lymphatic system disorders | 0 | - | - | - | - | 1 | - | 0 |
|  |  | Eye disorders | 0 | - | - | - | - | 1 | - | 1 |
|  |  | Gastrointestinal disorders | 8 | - | - | - | - | 3 | - | 4 |
|  |  | General disorders and administration site conditions | 3 | - | - | - | - | 6 | - | 9 |
|  |  | Immune system disorders | 0 | - | - | - | - | 1 | - | 2 |
|  |  | Infections and infestations | 2 | - | - | - | - | 1 | - | 0 |
|  |  | Injury, poisoning and procedural complications | 1 | - | - | - | - | 6 | - | 1 |
|  |  | Investigations | 1 | - | - | - | - | 2 | - | 0 |
|  |  | Musculoskeletal and connective tissue disorders | 2 | - | - | - | - | 10 | - | 5 |
|  |  | Neoplasms benign, malignant and unspecified (inclcysts and polyps) | 0 | - | - | - | - | 0 | - | 1 |
|  |  | Nervous system disorders | 1 | - | - | - | - | 4 | - | 5 |
|  |  | Psychiatric disorders | 0 | - | - | - | - | 0 | - | 2 |
|  |  | Respiratory, thoracic and mediastinal disorders | 0 | - | - | - | - | 3 | - | 0 |
|  |  | Skin and subcutaneous tissue disorders | 0 | - | - | - | - | 2 | - | 1 |
| Yoshino et al. 2021[15] | 25 | Any AEs | 0 | 0 | - | - | - | - | - | - |

Abbreviations: AE: adverse event; N: sample size; SAE: serious adverse event. *Number of participants.

**Supplementary Figure 1. Forest plot of the effect of NMN supplement on BMI, blood pressure and markers of liver function.**


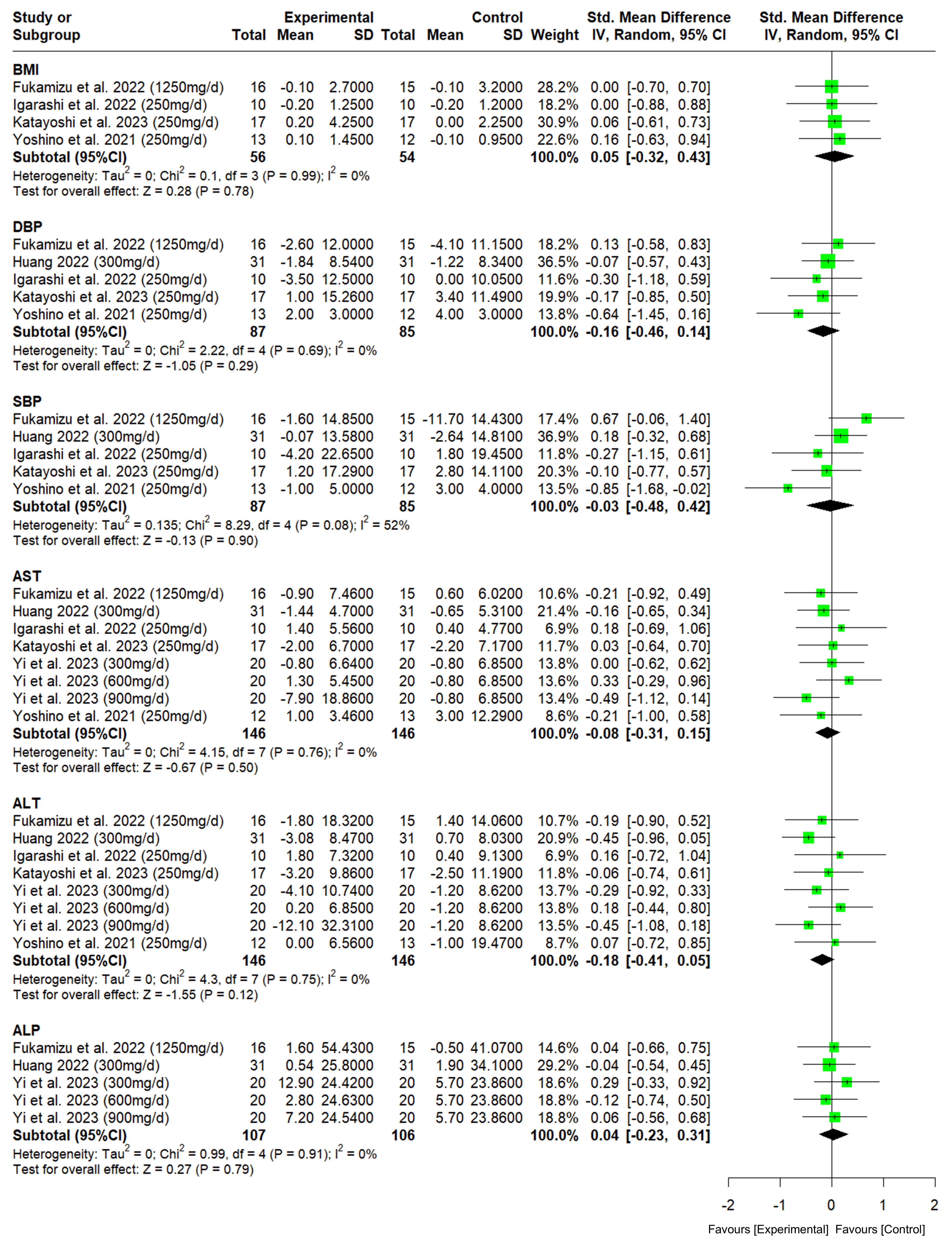

Supplement: Supplementary file 1 — Supplementary file1 (DOCX 10.1 MB) [file 11892_2024_1557_MOESM1_ESM.docx]
